# Avian Influenza A(H7N9) Virus Antibodies in Close Contacts of Infected Persons, China, 2013–2014

**DOI:** 10.3201/eid2104.141442

**Published:** 2015-04

**Authors:** Mai-Juan Ma, Guang-Yuan Ma, Xiao-Xian Yang, Shan-Hui Chen, Gregory C. Gray, Teng Zhao, Jing Bao, Jing-Jing Zhou, Yan-Hua Qian, Bin Lu, Xia Ling, Wu-Chun Cao

**Affiliations:** Beijing Institute of Microbiology and Epidemiology, Beijing, China (M.-J. Ma, W.-C. Cao);; Wuxi Center for Disease Control and Prevention, Wuxi, China (G.-Y. Ma, S.-H. Chen, J. Bao, Y.-H. Qian, B. Lu, X. Ling);; State Key Laboratory of Pathogen and Biosecurity, Beijing (X.-X. Yang, T. Zhao, J.-J. Zhou);; School of Public Health, Central South University, Changsha, China (X.-X. Yang);; Duke University Medical Center, Durham, North Carolina, USA (G.C. Gray)

**Keywords:** subtype H7N9 virus, influenza A(H7N9) virus, H7N9, influenza, avian influenza, viruses, human-to-human transmission, human, close contacts, seroepidemiology, China

**To the Editor:** From early 2013 (*1*) through November 2014, >460 human cases of laboratory-confirmed avian influenza A(H7N9) virus infection occurred in China. Although human-to-human transmission of subtype H7N9 virus is not common, evidence has been reported of probable transmission among several family clusters (*2*), between 2 household contacts (*3*), and between a doctor and an infected patient (*4*). Taken together, these observations suggest that family members, health care providers, and other close contacts (hereafter called contacts) of H7N9-infected persons may be at risk for infection.

In China, national guidelines regarding H7N9-infected patients call for observation of contacts for 7 days after exposure for signs and symptoms of infection and, if any occur, collection of throat swab specimens for testing by molecular assays (*5*). The guidelines do not call for serologic testing. Because human avian influenza infections may be mild or asymptomatic, we sought to determine whether serologic testing would show evidence of H7N9 virus infection among contacts of infected persons during the 2013–2014 epidemic in China. Contacts were defined in accordance with China’s guidelines for prevention and control of human H7N9 virus infection (*5*,*6*). The institutional review board of Wuxi Center for Disease Control and Prevention, Wuxi, Jiangsu Province, China, reviewed and approved this study.

During the epidemic, we recruited contacts of patients in Wuxi and collected throat swab specimens when signs or symptoms of infection developed; serum samples were collected 2–3 weeks later. Swab specimens were tested for H7N9 virus by using real-time reverse transcription PCR (*7*). Serum samples were tested for antibodies against hemagglutinin antigens of 3 avian influenza viruses (A/Anhui/1/2013 [H7N9], A/Anhui/1/2005 [H5N1]-RG5, and A/chicken/Jiangsu/1/00 [H9N2]) (*8*) by using a horse erythrocyte hemagglutination inhibition (HI) assay and against the hemagglutinin antigens of 2 seasonal influenza viruses (A/California/07/2009 [H1N1] and A/Victoria/210/2009 [H3N2]) by using a turkey erythrocyte HI assay. Serum samples with HI titers >1:40 against H7N9 virus were confirmed positive by microneutralization assay.

Ten laboratory-confirmed human infections with H7N9 virus occurred in Wuxi during March 29, 2013–May 15, 2014. In total, 225 contacts of 7 H7N9-infected patients were enrolled in the study (Table); contacts included 30 family members; 177 health care workers (54 physicians, 119 nurses who provided patient care with standard precautions, 2 hospital attendants, and 2 nurse assistants who provided services related to patient care, safety, and comfort, including anxiety relief, and medical observation); and 18 other contacts (8 friends who visited the patient in the hospital, 2 patients who shared the same room, and 8 patients who shared the same hospital area). The contacts of 3 other H7N9-infected patients declined to participate in the study.

**Table:** Demographic characteristics and HI antibody titers against influenza subtype H7N9, H5N1, H9N2, H1N1, and H3N2 viruses among close contacts of avian influenza A(H7N9)–infected persons, China, 2013–2014*

Characteristics	Close contacts, N = 225		
	Family members, n = 30	Health care workers, n = 177	Others, n = 18
Mean age, y ± SD	48.03 ± 17.79	33.71 ± 7.97	68.50 ± 14.89
Sex			
F	18 (60.0)	135 (76.3)	4 (22.2)
M	12 (40.0)	42 (23.7)	14 (77.8)
Exposure duration, mean days ± SD	7.38 ± 4.70	4.42 ± 3.67	3 ± 1.48
Virus subtype and HI titer†			
H7N9			
<1:80	29 (96.7)	173 (97.7)	18 (100.0)
>1:80	1 (3.3)	4 (2.3)	0
H5N1			
<1:80	30 (100.0)	177 (100.0)	20 (100.0)
>1:80	0	0	0
H9N2			
<1:80	30 (100.0)	177 (100.0)	20 (100.0)
≥1:80	0	0	0
H1N1			
<1:80	30 (100.0)	172 (97.1)	18 (100.0)
>1:80	0	5 (2.9)	0
H3N2			
<1:80	20 (66.7)	89 (50.3)	9 (50.0)
>1:80	10 (33.3)	88 (49.7)	9 (50.0)
MN titer, H7N9‡			
<1:10	0	6 (35.3)	1 (100.0)
1:10	0	4 (23.5)	0
1:20	3 (75.0)	3 (17.6)	0
1:40	0	3 (17.6)	0
1:80	1 (25.0)	1 (5.9)	0

*Data are no. (%) unless otherwise indicated. A comparison of HI titers for control serum samples against reference influenza virus strains used in this study is shown in the online Technical Appendix (http://wwwnc.cdc.gov/EID/article/21/4/14-1442-Techapp1.pdf). HI, hemagglutination inhibition; MN, microneutralization assay. †HI titer cut points were selected conservatively at >1:80 on the basis of World Health Organization recommendations for human infection with influenza A(H5N1) virus (http://www.who.int/influenza/resources/documents/RecAIlabtestsAug07.pdf). ‡Results for 22 close contacts (17 health care workers, 4 family members, and 1 other close contact) with an HI titer of >1:40.

Serologic assay results showed that, 14–28 days after their earliest exposure to an H7N9-infected patient, 22 (9.8%) contacts had elevated HI antibody titers (>1:40) against H7N9 virus; titers were 1:40 for 17 contacts and 1:80 for 5 contacts. Positive results for all 22 serum samples were validated by microneutralization assay; 15 (68.2%) samples had microneutralization antibody titers of >1:10 against H7N9 virus antigen (Table). Of the contacts with an HI titer of >1:80 and microneutralization titer of >1:40, 3 were nurses, 1 was a nurse assistant, and 1 was a family member (a patient’s daughter). All 5 of these contacts had antibody titers of <1:40 to influenza subtype H1N1, H5N1, and H9N2 viruses, and 2 of the nurses had HI antibody titers of 1:80 against subtype H3N2 virus. All contacts denied having influenza-like respiratory symptoms during the 28 days of follow-up and also denied recent exposure to poultry or pigs or their environments. Of contacts with an HI titer of >1:80 to seasonal H1N1 virus, 3 had titer of 1:80, and 1 each had titer of 1:160 or 1:640. Of the 225 contacts, 108 had HI titers >1:80 against seasonal H3N2 virus (1:80 for 63 contacts, 1:160 for 27 contacts, 1:320 for 9 contacts, and >1:640 for 8 contacts). All contacts had influenza subtype H5N1 and H9N2 antibody titers of <1:80.

A previous epidemiologic study (*2*) reported the medical monitoring of 2,657 contacts of H7N9-infected patients in mainland China and found that, for 28 of the contacts, respiratory symptoms developed within 7 days after monitoring began. Results of molecular assay testing of throat swab specimens for H7N9 virus were negative for all 28 contacts; the study did not include serologic testing. However, small serologic survey studies in Taiwan (*9*) and household contacts in mainland China (*10*) showed no evidence of human-to-human transmission among contacts.

A limitation of our study is that we did not collect serum samples from all contacts of infected persons or from controls; therefore, we could not assess the possibility of false-positive results or asymptomatic infections. However, our findings of elevated levels of subtype H7N9 antibody among 6.7% of contacts during this epidemic in China offer evidence that human-to-human transmission of H7N9 virus may occur among contacts of infected persons.

Technical Appendix Cross-reaction of hemagglutination inhibition titers of control serum samples to virus strains used in this study.
