# Supplementary material for: Avian Influenza A(H7N9) Virus Antibodies in Close Contacts of Infected Persons, China, 2013–2014
Source: Emerg Infect Dis. 2015 Apr;21(4):709–11. doi: 10.3201/eid2104.141442 (PMC4378467; doi:10.3201/eid2104.141442)
Supplement: Technical Appendix — Cross-reaction of hemagglutination inhibition titers of control serum samples to virus strains used in this study. [file 14-1442-Techapp-s1.pdf]

Article DOI: http://dx.doi.org/10.3201/eid2104.141442

Avian Influenza A(H7N9) Virus Antibodies in Close Contacts of Infected Persons, China, 2013–2014

Technical Appendix

Technical Appendix Table. Comparison of hemagglutination inhibition titers for control sera against reference influenza virus strains used in this study.

	Titer, by reference influenza A virus subtype				
Antisera control	H7N9	H5N1	H9N2	H1N1	H3N2
H7N9*	1:320	<1:10	<1:10	<1:10	<1:10
A/Anhui/01/05 (Avian H5N1 virus)†	<1:10	1:2560	<1:10	1:40	<1:10
H9N2*	<1:10	<1:10	1:80	<1:10	1:20
A/Sichuan/SWL1/2009 (Human H1N1 virus)†	<1:10	<1:10	1:20	1:1280	<1:10
A/ Fujian Tongan/196/2009 (Human H3N2 virus)†	<1:10	<1:10	1:40	1:20	1:1280

*Antisera were purchased from the Harbin Weike Biotechnology Development Company subordinated Harbin Veterinary research institute, Chinese Academy of Agricultural Sciences (Harbin, China).
†Sheep antisera.
